# Therapeutic effects of LASSBio-596 in an elastase-induced mouse model of emphysema

**DOI:** 10.3389/fphys.2015.00267

**Published:** 2015-09-30

**Authors:** Gisele A. Padilha, Isabela Henriques, Miquéias Lopes-Pacheco, Soraia C. Abreu, Milena V. Oliveira, Marcelo M. Morales, Lidia M. Lima, Eliezer J. Barreiro, Pedro L. Silva, Debora G. Xisto, Patricia R. M. Rocco

**Affiliations:** ^1^Laboratory of Pulmonary Investigation, Carlos Chagas Filho Institute of Biophysics, Federal University of Rio de JaneiroRio de Janeiro, Brazil; ^2^Laboratory of Cellular and Molecular Physiology, Carlos Chagas Filho Institute of Biophysics, Federal University of Rio de JaneiroRio de Janeiro, Brazil; ^3^Laboratory of Evaluation and Synthesis of Bioactive Substances, Federal University of Rio de JaneiroRio de Janeiro, Brazil

**Keywords:** emphysema, remodeling, inflammation, macrophage, elastic fiber

## Abstract

Emphysema is an intractable pulmonary disease characterized by an inflammatory process of the airways and lung parenchyma and ongoing remodeling process in an attempt to restore lung structure. There is no effective drug therapy that regenerates lung tissue or prevents the progression of emphysema; current treatment is aimed at symptomatic relief. We hypothesized that LASSBio-596, a molecule with potent anti-inflammatory and immunomodulatory effects, might reduce pulmonary inflammation and remodeling and thus improve lung function in experimental emphysema. Emphysema was induced in BALB/c mice by intratracheal administration of porcine pancreatic elastase (0.1 IU) once weekly during 4 weeks. A control group received saline using the same protocol. After the last instillation of saline or elastase, dimethyl sulfoxide, or LASSBio-596 were administered intraperitoneally, once daily for 8 days. After 24 h, in elastase-induced emphysema animals, LASSBio-596 yielded: (1) decreased mean linear intercept, hyperinflation and collagen fiber content, (2) increased elastic fiber content, (3) reduced number of M1 macrophages, (4) decreased tumor necrosis factor-α, interleukin-1β, interleukin-6, and transforming growth factor-β protein levels in lung tissue, and increased vascular endothelial growth factor. These changes resulted in increased static lung elastance. In conclusion, LASSBio-596 therapy reduced lung inflammation, airspace enlargement, and small airway wall remodeling, thus improving lung function, in this animal model of elastase-induced emphysema.

## Introduction

Emphysema, defined as irreversible destruction of the alveoli, is associated with an inflammatory process of the airways and lung parenchyma (Newell, 2008) and an ongoing remodeling process in an attempt to restore lung structure (Papaioannou et al., 2010). Decades of research have still not resulted in an effective treatment other than cessation of cigarette smoking, a highly addictive behavior. Strategies focusing on development of anti-inflammatory drugs, such as antagonists of cytokines, including TNF-α (Dentener et al., 2008) and interleukin (IL)-8 (Mahler et al., 2004), have proved disappointing, and current treatment is aimed at symptomatic relief. One potential therapeutic approach for emphysema is decreasing the chronic inflammation and fibrogenesis associated with induction of lung repair and regeneration.

LASSBio-596 is an achiral molecule in the carbamoyl-benzoic acid class that was designed as a symbiotic agent from the hybridization of prototypes originating from thalidomide, arylsulfonamide, and sildenafil (Lima et al., 2002; Rocco et al., 2010). The therapeutic potential of LASSBio-596 has been studied in experimental models of acute lung inflammation induced by *Escherichia coli* lipopolysaccharide (Rocco et al., 2003), microcystin-LR (Carvalho et al., 2010), and chronic allergic inflammation (Campos et al., 2006); it has been found to act mainly on inflammatory processes, improving pulmonary function. Furthermore, LASSBio-596 is not associated with relevant side effects, unlike corticosteroids, which are commonly used in emphysema (Calverley, 2014). Therefore, we hypothesized that, in an elastase-induced emphysema model, LASSBio-596 therapy might act on lung inflammation and remodeling as well as stimulate elastogenesis.

## Materials and methods

This study was approved by the Animal Research Ethics Committee of the Federal University of Rio de Janeiro Health Sciences Center (CEUA-CCS-IBCCF 019). All animals received humane care in compliance with the “Principles of Laboratory Animal Care” formulated by the National Society for Medical Research and the “Guide for the Care and Use of Laboratory Animals” prepared by the U.S. National Academy of Sciences.

### Animal preparation and experimental protocol

**Figure 1:**
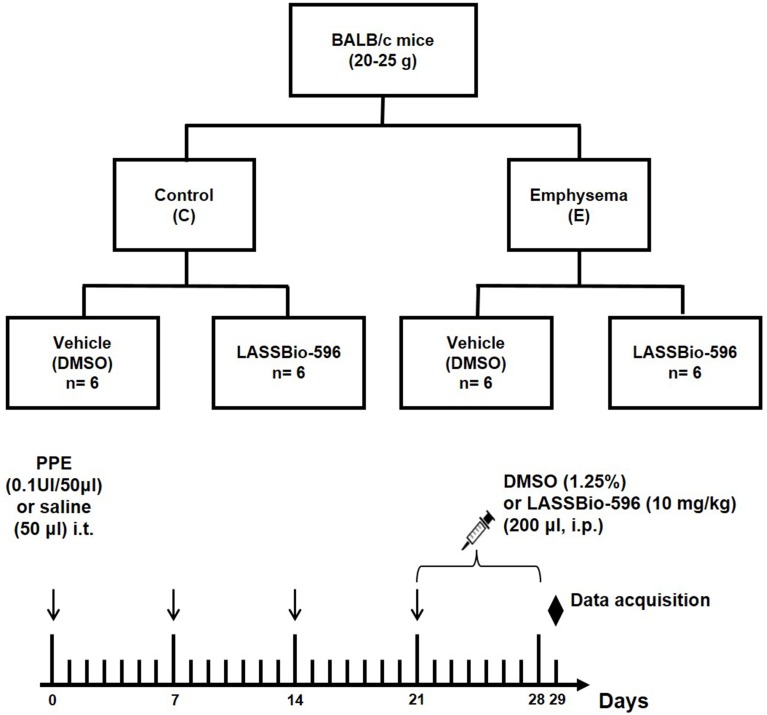
**Flowchart and timeline of study design**. Control (C), four intratracheal administrations of saline; Emphysema (E), four intratracheal administrations of porcine pancreatic elastase (PPE); DMSO, vehicle (dimethyl sulfoxide)—intraperitoneal treatment with DMSO for 8 days; LASSBio-596, intraperitoneal treatment with LASSBio-596 for 8 days. All data were analyzed on day 29.

Thirty-six male BALB/c mice (weight 20–25 g, age 8–10 weeks) were used in this study. The animals were kept under specific pathogen-free conditions in the animal care facility of the Laboratory of Pulmonary Investigation, Federal University of Rio de Janeiro. All animals were randomly assigned to two groups. In control (C) animals, saline was instilled intratracheally (50 μl), whereas emphysema (E) mice received porcine pancreatic elastase (PPE, Sigma Chemical Co., St. Louis, MO, USA) (0.1 UI in 50 μl saline solution) via the same route. Saline and PPE were injected intratracheally once a week for 4 weeks. For intratracheal instillation, mice were anesthetized with sevoflurane. Induction of emphysema was performed according to a previous protocol established in our laboratory (Cruz et al., 2012). Static lung elastance (Est,L), mean linear intercept (Lm), and mononuclear cells and neutrophils (tissue cellularity) were analyzed at day 21 in a total of 12 animals (*n* = 6/group). The remaining 24 animals were subjected to the same protocol, but, after day 21, were further randomized into subgroups to receive DMSO (10 mg/kg, 0.02 mL, intraperitoneally [i.p.]) or LASSBio-596 (596, 10 mg/kg, 0.02 mL i.p.) for 8 consecutive days (Figure 1). These animals were analyzed on day 29.

### Lung mechanics

Twenty-four hours after the last intraperitoneal treatment, animals were sedated (diazepam 1 mg i.p.), anesthetized (thiopental sodium 20 mg/kg i.p.), tracheotomized, paralyzed (vecuronium bromide 0.005 mg/kg i.v.), and ventilated with a constant flow ventilator (Samay VR15; Universidad de la Republica, Montevideo, Uruguay) set to the following parameters: frequency 100 breaths/min, tidal volume (V_*T*_) 0.2 mL, and fraction of inspired oxygen (FiO_2_) 0.21. After neuromuscular blockade, adequate depth of anesthesia was assessed by evaluating pupil size and reactivity to light (Correa et al., 2001). The anterior chest wall was surgically removed and a positive end-expiratory pressure (PEEP) of 2 cm H_2_O was applied. After a 10 min ventilation period, airflow, volume, and tracheal pressure (Ptr) were measured. In an open chest preparation, Ptr reflects transpulmonary pressure (PL). Est,L was measured by the end-inflation occlusion method (Bates et al., 1985). All mechanical data were analyzed using the ANADAT data analysis software (RHT-InfoData, Inc., Montreal, Quebec, Canada). At the end of the experiments (20 min), animals were euthanized and lungs prepared for histology and protein analysis.

### Lung histology

Immediately after assessment of lung mechanics, a laparotomy was performed and heparin (1000 IU) was injected into the inferior vena cava. The trachea was clamped at end-expiration (2 cm H_2_O) and the abdominal aorta and vena cava were sectioned, yielding a massive hemorrhage that quickly killed the animals. The left lung was then removed, frozen quickly by immersion in liquid nitrogen, fixed with Carnoy's solution, and embedded in paraffin. Three 4-μm-thick slices were cut from each lung and stained with hematoxylin-eosin for lung morphometric analysis, Weigert's resorcin-fuchsin method with oxidation for quantification of elastic fibers in alveolar septa, or Sirius red solution for quantification of collagen fibers in alveolar septa (Fullmer et al., 1974; Montes, 1996). Lung morphometry analysis was performed using a reticle composed of a grid with 100 points and 50 lines of known length coupled to the integrating eyepiece of a conventional light microscope (Olympus BX51,Olympus Latin America-Inc., Brazil). The volume fractions of the lung occupied by collapsed alveoli (alveoli with rough or plicate walls), normal pulmonary areas, or hyperinflated structures (alveolar ducts, alveolar sacs, or alveoli, all with maximal chord length in air >120 μm) were determined by the point-counting technique (Weibel, 1990) across 10 random, non-coincident microscopic fields. Briefly, points falling on collapsed, normal pulmonary areas or hyperinflated structures were counted and divided by the total number of points in each microscopic field. Enlargement of air spaces was evaluated using mean linear intercept measurement (Lm) (Dunnill, 1964). The number of total cells, neutrophils, and mononuclear cells, as well as the amount of pulmonary tissue, were also determined by the point-counting technique across 10 random, non-coincident microscopic fields at × 1000 magnification. Data were reported as the fraction area of pulmonary tissue.

### Collagen and elastic fibers quantification

Collagen (Picrosirius-polarization method) and elastic fibers were quantified in alveolar septa at × 400 magnification. The area occupied by fibers was determined by digital densitometric recognition (Image-Pro Plus 7.1 Software, Media Cybernetics—Silver Spring, MD, USA) and divided by the area of each studied septum. The results were expressed as the fractional area occupied by elastic and collagen fibers in the alveolar septa. Bronchi and blood vessels were excluded from the measurements to avoid biased results.

### Immunohistochemistry for M1 and M2 macrophages

Immunohistochemical analysis for M1 and M2 macrophages in lung tissue was performed using inducible nitric oxide synthase rabbit anti-mouse polyclonal antibody (catalog no. RB-9242, Thermo Scientific) and arginase-1 rabbit anti-mouse polyclonal antibody (catalog no. SC-20150, Santa Cruz Biotechnology), respectively. Paraffin-embedded tissue sections (4 μm) were dewaxed, rehydrated, and underwent heat-mediated antigen retrieval. Antibodies were detected with a secondary antibody labeled with peroxidase from Nichirei Biosciences (Tokyo, Japan) (Histofine mouse MAX PO anti-rabbit), followed by the chromogen substrate diaminobenzidine (liquid DAB; catalog no. K3468, DakoCytomation, USA). Slides were counterstained with hematoxylin-eosin. Analysis was performed in 30 images, at magnification × 400, under a light microscope (Olympus BX51, Olympus Latin America-Inc., Brazil). The areas occupied by cells with positive staining for the phenotype marker were then measured and divided by tissue area using Image-Pro Plus 6.3 for Windows software (Media Cybernetics, Silver Spring, MD, USA) and expressed as fraction area occupied by positive cells (Lopes-Pacheco et al., 2013).

### Enzyme-linked immunosorbent assay

Central slices of right lung were cut and flash-frozen. IL-1β, tumor necrosis factor (TNF)-α, IL-6, tumor growth factor (TGF)-β, and vascular endothelial growth factor (VEGF) were quantified by enzyme-linked immunosorbent assay (ELISA, Duo Set; R & D Systems, Minneapolis, USA), according to manufacturer protocols.

### Statistical analysis

Data were tested for normality using the Kolmogorov-Smirnov test with Lilliefors correction, whereas the Levene median test was used to evaluate the homogeneity of variances. Student's *t*-test was used to compare differences between control and emphysema animals at day 21. Two-Way ANOVA followed by Tukey's test was used to compare control and emphysema groups treated with DMSO or LASSBio-596. For non-parametric data, One-Way ANOVA on ranks followed by Dunn's *post-hoc* test was selected. The significance level was always set at 5%. Parametric data were expressed as mean ± standard deviation (SD), while non-parametric data were expressed as median (interquartile range). All tests were performed in the GraphPad Prism statistical software (version 5.00, GraphPad Software, La Jolla, California, USA).

## Results

**Figure 2:**
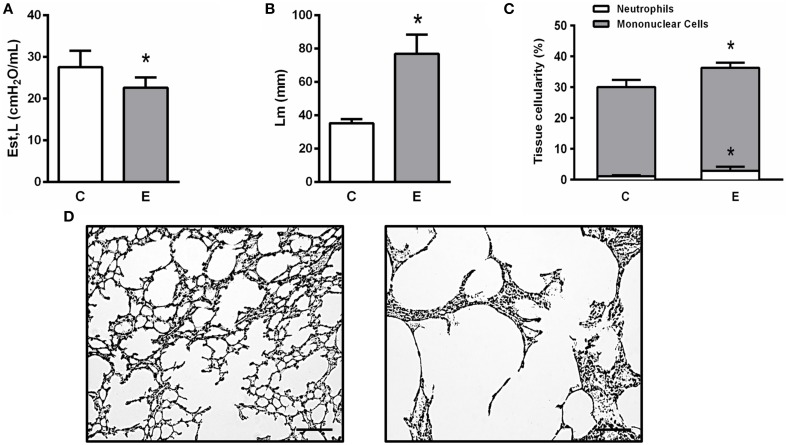
**Static lung elastance (Est,L) (A), mean linear intercept (Lm) (B), mononuclear cells, neutrophils, and total cells in lung tissue (C), and representative photomicrographs of lung parenchyma stained with hematoxylin-eosin**. Note enlargement of alveolar space in the Egroup (arrow) **(D)**. Presence of pulmonary emphysema on day 21 was analyzed in two groups **(C,E)**. C, mice received intratracheal saline and were analyzed on day 21; E, mice received intratracheal porcine pancreatic elastase and were analyzed on day 21. ^*^Significantly different from C (*p* < 0.05).

At day 21, Est,L was significantly lower in E compared to C animals (Figure 2A). Histological analysis revealed a higher Lm (Figure 2B) as well as a greater number of mononuclear cells and neutrophils in lung tissue in E than C (Figure 2C). Figure 2D shows destruction of alveolar septa (black arrow), associated with increased cell infiltration.

**Figure 3:**
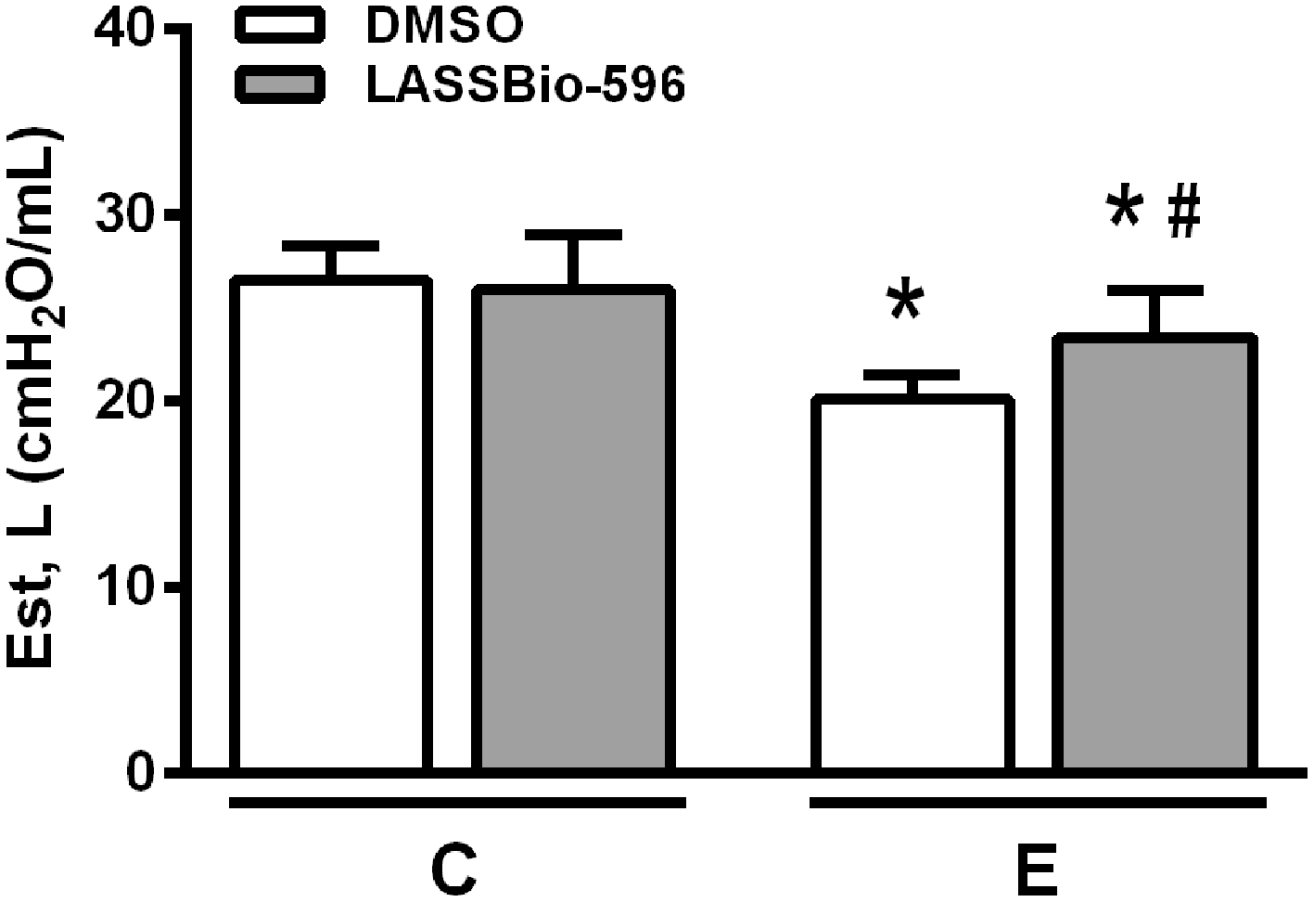
**Static lung elastance (Est,L)**. Values are means (± SD) of six animals in each group. C-DMSO: mice received intratracheal saline and were treated with DMSO. C-LASSBio-596 mice received intratracheal saline and were treated with LASSBio-596. E-DMSO: mice received intratracheal porcine pancreatic elastase and were treated with DMSO. E-LASSBio-596: mice received intratracheal porcine pancreatic elastase and were treated with LASSBio-596. ^*^Significantly different from C (*p* < 0.05). ^#^Significantly different from E-DMSO (*p* < 0.05).

After confirmation of emphysema features on day 21, mice received DMSO or LASSBio-596 and lung mechanics were analyzed at day 29. Est,L was lower in the E-DMSO group compared to C-DMSO. E-LASSBio-596 animals exhibited higher static lung elastance compared to E-DMSO, but did not achieve control (C) values (Figure 3).

**Figure 4:**
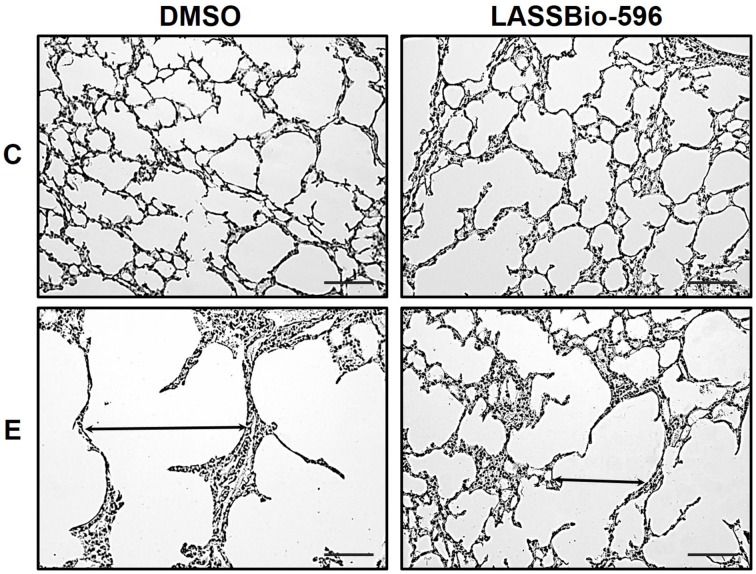
**Representative photomicrographs of lung parenchyma stained with hematoxylin-eosin**. Note the enlargement of alveolar space in the E-DMSO group (arrow). C-DMSO: mice received intratracheal saline and were treated with DMSO. C-LASSBio-596 mice received intratracheal saline and were treated with LASSBio-596. E-DMSO mice received intratracheal porcine pancreatic elastase and were treated with DMSO. E-LASSBio-596: mice received intratracheal porcine pancreatic elastase and were treated with LASSBio-596. Original magnification: × 200. Bars = 100 μm.

**Figure 5:**
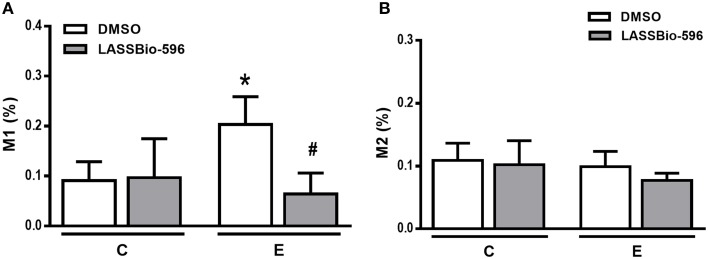
**Immunohistochemical analysis for the M1 (A) and (B) M2 macrophage phenotypes in lung tissue**. Values are means (± SD) of six animals in each group. C-DMSO: mice received intratracheal saline and were treated with DMSO. C-LASSBio-596 mice received intratracheal saline and were treated with LASSBio-596. E-DMSO: mice received intratracheal porcine pancreatic elastase and were treated with DMSO. E-LASSBio-596: mice received intratracheal porcine pancreatic elastase and were treated with LASSBio-596. ^*^Significantly different from respective group C (*p* < 0.05). ^#^Significantly different from E-DMSO (*p* < 0.05).

E-DMSO animals exhibited higher Lm compared to C-DMSO, and this parameter was reduced in E-LASSBio-596 (Figure 4). Additionally, E-DMSO animals showed areas of hyperinflation characteristic of emphysema, which were reduced after therapy with LASSBio-596 (Table 1). The number of neutrophils was higher in E-DMSO and E-LASSBio-596 animals compared to the respective control groups, but no difference was observed between E-DMSO and E-LASSBio-596. Moreover, the number of mononuclear cells did not differ among groups (Table 1). Even though no differences were observed in the number of mononuclear cells, M1 macrophage subpopulations increased in E-DMSO compared to C-DMSO. E-LASSBio-596 mice showed a reduction in the percentage of M1 compared to E-DMSO (Figure 5A). On the other hand, no differences in M2 subpopulations were observed among groups (Figure 5B).

**Table 1:** **Lung histology**.

**Groups**	**C**		**E**	
	**DMSO**	**LASSBio-**	**DMSO**	**LASSBio-**
		**596**		**596**
Normal (%)	94.6±1.7	93.4±1.8	51.4±19.1^*^	75.9±6.1^*,#^
Collapse (%)	5.4±1.7	6.2±2.0	4.9±5.9	3.2±1.2
Hyperinflation (%)	0.0±0.0	0.4±0.8	43.6±13.8^*^	21.0±6.2^*,#^
Mean linear intercept (Lm)	42.2±3.8	43.5±5.3	59.4±7.6^*^	50.7±6.6^*,#^
Mononuclear cells (%)	28.5±1.6	30.0±1.6	25.7±1.0	27.3±1.7
Neutrophils (%)	2.9±0.4	3.0±0.3	5.2±0.6^*^	4.8±0.4^*^

All data were collected in 10 random, non-coincident fields per mice. Values are means (± SD) of six animals in each group. C-DMSO: mice received intratracheal saline and were treated with DMSO. C-LASSBio-596 mice received intratracheal saline and were treated with LASSBio-596. E-DMSO: mice received intratracheal porcine pancreatic elastase and were treated with DMSO. E-LASSBio-596: mice received intratracheal porcine pancreatic elastase and were treated with LASSBio-596. ^*^Significantly different from respective group C (p < 0.05). ^#^Significantly different from E-DMSO (p < 0.05).

**Figure 6:**
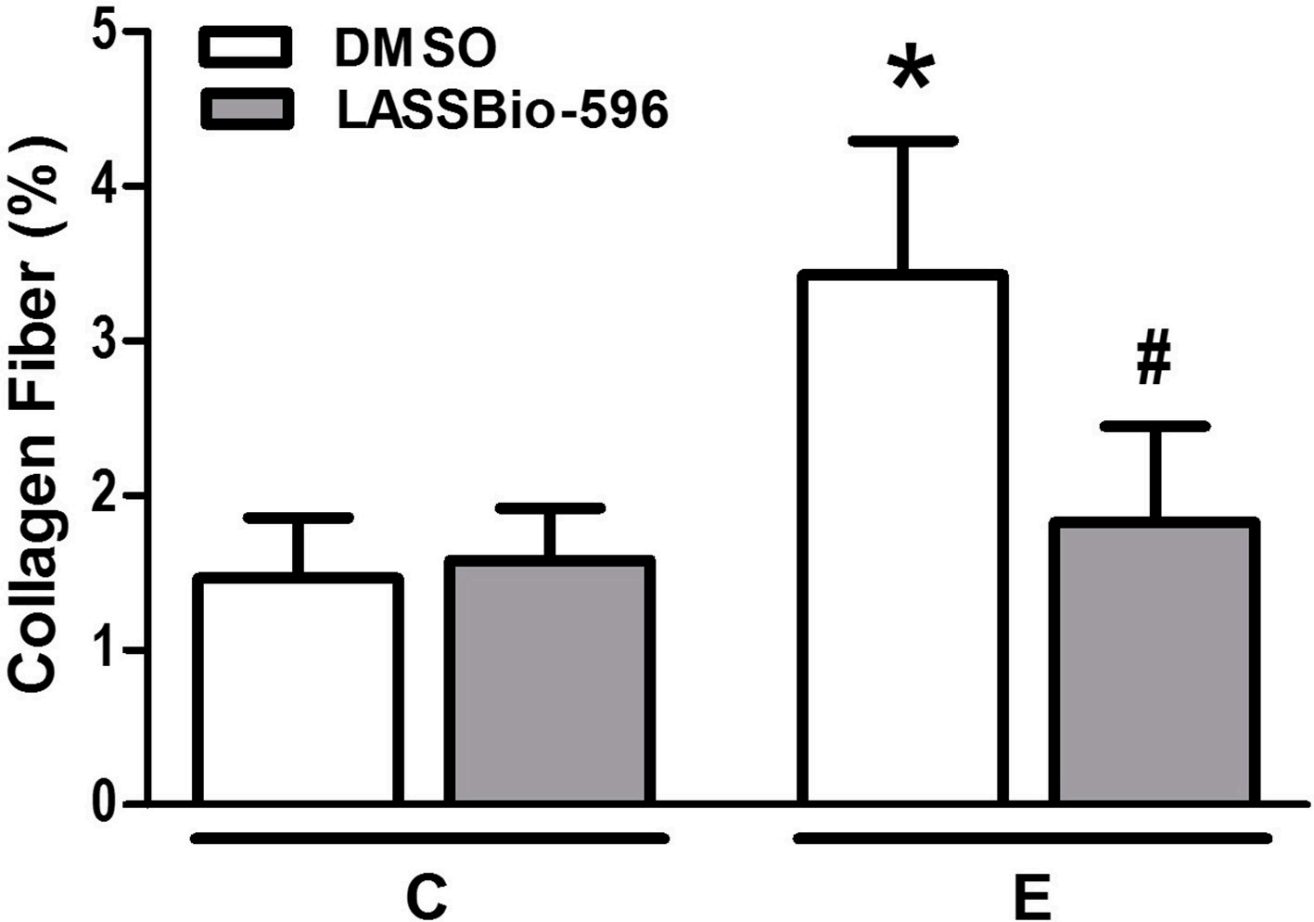
**Collagen fiber content in lung parenchyma**. All data were collected in 10 random, non-coincident fields per mouse. Values are means (± SD) of six animals in each group. C-DMSO: mice received intratracheal saline and were treated with DMSO. C-LASSBio-596 mice received intratracheal saline and were treated with LASSBio-596. E-DMSO: mice received intratracheal porcine pancreatic elastase and were treated with DMSO. E-LASSBio-596: mice received intratracheal porcine pancreatic elastase and were treated with LASSBio-596. ^*^Significantly different from respective group C (*p* < 0.05). ^#^Significantly different from E-DMSO (*p* < 0.05).

**Figure 7:**
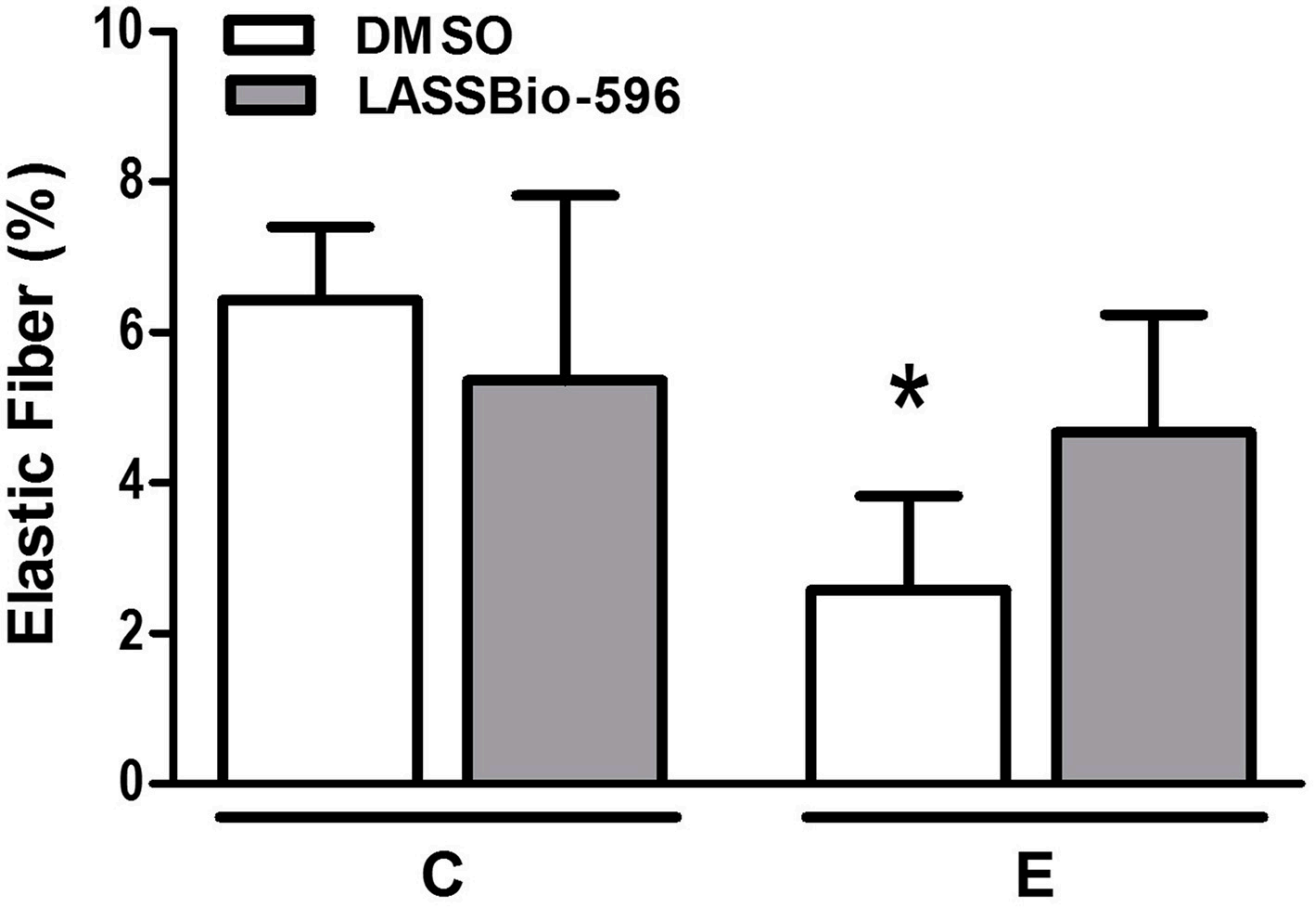
**Elastic fiber content in lung parenchyma**. All data were collected in 10 random, non-coincident fields per mouse. Values are means (± SD) of six animals in each group. C-DMSO: mice received intratracheal saline and were treated with DMSO. C-LASSBio-596 mice received intratracheal saline and were treated with LASSBio-596. E-DMSO: mice received intratracheal porcine pancreatic elastase and were treated with DMSO. E-LASSBio-596: mice received intratracheal porcine pancreatic elastase and were treated with LASSBio-596. ^*^Significantly different from respective group C (*p* < 0.05).

The content of collagen fibers in E-DMSO mice was higher compared to C-DMSO. E-LASSBio-596 mice showed a lower collagen fiber content than E-DMSO (Figure 6). Furthermore, elastic fiber content was lower in the E-DMSO group compared to C-DMSO, and higher in E-LASSBio-596 than in E-DMSO (Figure 7).

**Figure 8:**
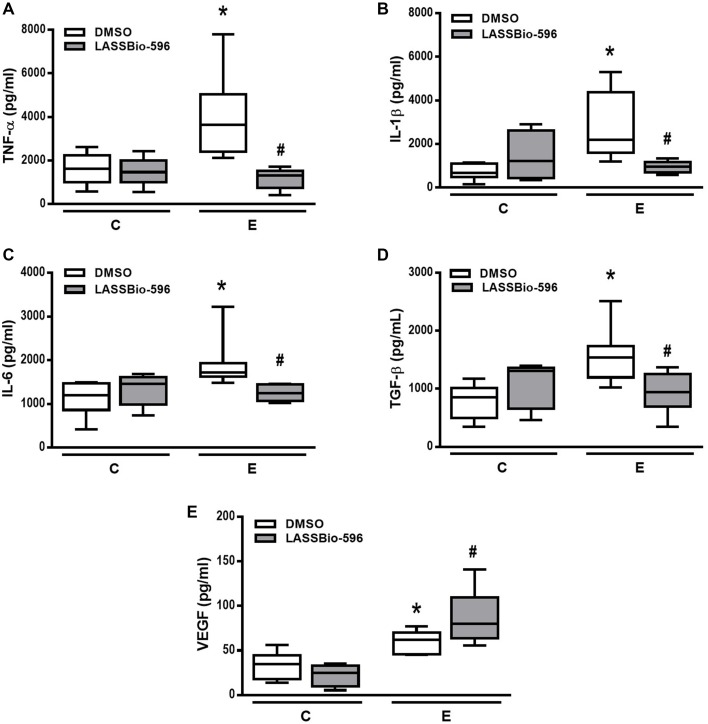
**Levels of tumor necrosis factor (TNF)-α (A), interleukin (IL)-1β (B), IL-6 (C), transforming growth factor (TGF)-β (D) and vascular endothelial growth factor (VEGF) (E) quantified by ELISA in lung tissue**. Values are median (interquartile range) of six animals in each group. C-DMSO: mice received intratracheal saline and were treated with DMSO. C-LASSBio-596 mice received intratracheal saline and were treated with LASSBio-596. E-DMSO: mice received intratracheal porcine pancreatic elastase and were treated with DMSO. E-LASSBio-596: mice received intratracheal porcine pancreatic elastase and were treated with LASSBio-596. ^*^Significantly different from respective group C (*p* < 0.05). ^#^Significantly different from E-DMSO (*p* < 0.05).

The E-DMSO group showed increased levels of TNF-α, IL-1β, IL-6, TGF-β, and VEGF compared to C-DMSO. E mice treated with LASSBio-596 showed lower TNF-α, IL-1β, IL-6, and TGF-β levels compared to E-DMSO (Figures 8A–D). Interestingly, VEGF levels in E-LASSBio-596 were higher than in the E-DMSO group (Figure 8E).

## Discussion

In a murine model of elastase-induced pulmonary emphysema, we found that LASSBio-596: (1) improved elastic recoil, as observed by the decrease in hyperinflation and mean linear intercept; (2) decreased collagen fiber content and TGF-β levels in animals with experimental emphysema; (3) despite not acting on the percentage of total mononuclear cells, blunted the M1 phenotype; (4) reduced levels of TNF-α, IL1-β, IL-6 in lung tissue; and (5) increased VEGF levels in lung tissue. These results corroborated our hypothesis that treatment with LASSBio-596 acted on both the inflammatory and fibrogenic responses, as well as stimulated elastogenesis, leading to beneficial functional effects in animals with pulmonary emphysema.

Several animal models have been developed in attempts to reproduce pulmonary emphysema (Antunes and Rocco, 2011; Kurimoto et al., 2013). The model of multiple intratracheal instillations of PPE was initially developed by Lüthje et al. (2009) and modified by our group (Cruz et al., 2012). In contrast to the classical single-dose protocols of elastase-induced emphysema (Antunes and Rocco, 2011), which induce only emphysema-like lesions without systemic or cardiovascular impairment, the present model results in histological and ultrastructural lung changes and cardiac impairment that resemble human emphysema. In the present study, we evaluated lung morphofunctional changes associated with emphysema onset and start of therapy. At day 21, static lung elastance (Est,L) in the E group was reduced, which is one of the main features of pulmonary emphysema (GOLD, 2015). Interestingly, Est,L reduced from day 21 to 29, thus suggesting that emphysema worsens progressively over time. The loss of elastic recoil results from elastic fiber disruption in lung parenchyma, alveolar septa destruction (Takahashi et al., 2014) that leads to increased mean linear intercept (Figure 2B), and heterogeneous alveolar architecture after elastase instillation (Figure 2D) (Hamakawa et al., 2011). These lung histological changes were associated with an abnormal inflammatory response (Newell, 2008). In the alveolar surface, macrophage activation leads to the release of several inflammatory mediators and recruitment of neutrophils to the site of injury. In this line, we found that animals subjected to elastase instillation had a higher number of neutrophils in lung tissue (Bezerra et al., 2011).

Several studies have developed new pharmacologic strategies to mitigate the inflammation or remodeling processes (Churg et al., 2007; Baila et al., 2012). Despite the results obtained in animal models, application of these therapies in patients was associated with only mild improvement and did not prevent disease progression (Loza et al., 2012; Zuo et al., 2014). In our study, therapy with LASSBio-596 was started after identification of emphysema features, thus mimicking the clinical setting. LASSBio-596 is the result of molecular hybridization of thalidomide and arylsulfonamide derivatives (Lima et al., 2002) and has been shown to exert anti-inflammatory effects in both pulmonary (Rocco et al., 2003; Carvalho et al., 2010) and non-pulmonary diseases (Ribeiro et al., 2012). Our study was the first to analyze the effects of LASSBio-596 in elastase-induced emphysema. Emphysema animals treated with LASSBio-596 showed mild restoration of elastic recoil and a decreased mean linear intercept. We also observed reduced fibroproliferation, which may also have contributed to improvement of lung mechanics and morphometry. These beneficial effects of LASSBio-596 may be attributed to inhibition of NOX-2 and NOX-4, which are expressed in endothelial cells, macrophages, and myofibroblasts, and commonly related to cell proliferation, migration, and differentiation (van der Vliet, 2008; Griffith et al., 2009). In this line, even though NOX-2 and NOX-4 were not measured in the present study, LASSBio-596 has been shown to inhibit their mRNA expressions in hepatic injury after microcystin-Lr (Zin et al., 2013) and modulate NADPH oxidases, reducing the remodeling process (Ellmark et al., 2005).

LASSBio-596 did not alter the number of neutrophils in lung tissue, which was increased in our emphysema model. Even though the number of total mononuclear cells was not increased in lung tissue, we were unable to rule out that these changes might have been associated with differences in macrophage phenotypes (M1 and M2 macrophages) (Kunz et al., 2011). In this context, macrophage phenotypes in lung parenchyma were investigated. The classically activated M1 macrophages are associated with antimicrobial properties and the release of pro-inflammatory cytokines, whereas the alternatively activated M2 phenotype acts as an anti-inflammatory profile and contributes to wound healing (Lopes-Pacheco et al., 2013). In our study, M2 levels did not differ among groups; however, the levels of M1 macrophages were higher in E-DMSO compared to C-DMSO group. M1-derived cytokines, such as TNF-α, IL-1β, and IL-6, also play a role in the pathogenesis of COPD (Bucchioni et al., 2003; Sapey et al., 2009). In fact, we observed higher expression of these inflammatory proteins in E-DMSO compared to C-DMSO animals, and treatment with LASSBio-596 reduced M1 levels. High levels of IL-1β have been observed in bronchoalveolar lavage fluid both in experimental (Inoue et al., 2010) and in clinical settings (Ferhani et al., 2010), and may be inversely related to lung function. Similarly, we observed that LASSBio-596 reduced IL-1β levels in lung tissue, which were higher in E-DMSO than in C-DMSO mice. TNF-α is one of the most studied cytokines in the pathophysiology of COPD, and is associated with COPD exacerbations (Aaron et al., 2013). In the model of emphysema used in the present study, LASSBio-596 reduced TNF-α, which is consistent with previous studies in models of acute lung injury (Rocco et al., 2003) and asthma (Campos et al., 2006). IL-6, another mediator associated with worse prognosis in COPD patients (Celli et al., 2012; Ferrari et al., 2013), was reduced after LASSBio-596 therapy in the present study.

Growth factors are polypeptides involved in cellular transformation during remodeling process in several diseases (Barnes, 2008). In emphysema, the increase in TGF-β expression in epithelial cells and fibroblasts resulted in increased production of collagen and other extracellular matrix proteins (Takizawa et al., 2001; Takahashi et al., 2014). Similarly, in elastase-induced emphysema mice in this study, levels of TGF-β, and collagen fibers were increased, and LASSBio-596 treatment reduced both. We may hypothesize that LASSBio-596 acts on fibrogenesis not only by reducing TGF-β but also by decreasing lung inflammation and, thus, conversion of fibroblasts into myofibroblasts (Van Linthout et al., 2014). The increase in VEGF levels in our emphysema model was consistent with previous clinical investigations (Kranenburg et al., 2005). Previous studies in our lab have shown that the elastase-induced emphysema models is associated with pulmonary hypertension (Cruz et al., 2012), which is in line with enhanced VEGF expression (Tuder et al., 1994; Shehata et al., 1999). LASSBio-596 resulted in a further increase in VEGF, thus stimulating angiogenesis in an attempt to restore endothelial structure and function (Healy et al., 2000).

In parallel with the intense lung inflammatory process, emphysema is characterized by alveolar destruction and inadequate remodeling of the lung parenchyma (Suki and Bates, 2011), with both collagen fiber deposition and elastolysis. The resulting lung mechanical changes, which reduced Est,L, were very likely due to elastolysis, changes in the function of collagen fibers, and modifications in collagen organization (Ito et al., 2005). Emphysematous animals treated with LASSBio-596 showed an increase in elastic fiber content and a reduction in the amount of collagen fibers, in agreement with other experimental models of acute lung injury (Rocco et al., 2003) and asthma (Campos et al., 2006).

Some limitations of this study should be considered: (1) emphysema was induced by elastase, and the pathways involved in elastase induction differ from those occurring in smoke-induced emphysema, which may lead to differential responses to LASSBio-596; (2) a decrease in M1 macrophage levels was observed in emphysema group animals treated with LASSBio-596. Further studies should be performed to evaluate the role of M1 macrophages in the immunomodulating effect of LASSBio-596, e.g., by depletion of M1 macrophages in the mouse lung. Additionally, the role of LASSBio-596 needs to be analyzed in other cells, such as epithelial cells, endothelial cells, or fibroblasts; and (3) only a few specific cytokines and growth factors were evaluated; a wider range of mediators should be analyzed to provide a more complete understanding of the mechanisms associated with inflammation, apoptosis, and/or oxidative stress.

In conclusion, LASSBio-596 reduced lung inflammation and remodeling, improving lung function and structure, in an experimental model of elastase-induced pulmonary emphysema. These findings might be associated with the anti-inflammatory properties of LASSBio-596, modulating the M1 macrophage phenotype, and the release of cytokines and growth factors involved in the pathogenesis of emphysema. Nevertheless, further experimental studies are required to evaluate different mechanisms of LASSBio-596 in emphysema and thus reach clinical application.

## Author contributions

GP—interpretation of data for the work; drafting the work; and revising it for important intellectual content; final approval of the version to be published; agreement to be accountable for all aspects of the work in ensuring that questions related to the accuracy or integrity of any part of the work are appropriately investigated and resolved; IH Lucas—interpretation of data for the work, experimental design, and organization, data analyses; revised the work for important intellectual content; final approval of the version to be published, agreement to be accountable for all aspects of the work in ensuring that questions related to the accuracy or integrity of any part of the work are appropriately investigated and resolved; ML—data acquisition; revised the work for important intellectual content; final approval of the version to be published; agreement to be accountable for all aspects of the work in ensuring that questions related to the accuracy or integrity of any part of the work are appropriately investigated and resolved; SA—data acquisition; revised the work for important intellectual content; final approval of the version to be published; agreement to be accountable for all aspects of the work in ensuring that questions related to the accuracy or integrity of any part of the work are appropriately investigated and resolved; MO—data acquisition; revised the work for important intellectual content; final approval of the version to be published; agreement to be accountable for all aspects of the work in ensuring that questions related to the accuracy or integrity of any part of the work are appropriately investigated and resolved; MM—molecular biology analyses; revised the work for important intellectual content; final approval of the version to be published; agreement to be accountable for all aspects of the work in ensuring that questions related to the accuracy or integrity of any part of the work are appropriately investigated and resolved; LL—data analyses; revised the work for important intellectual content; final approval of the version to be published; agreement to be accountable for all aspects of the work in ensuring that questions related to the accuracy or integrity of any part of the work are appropriately investigated and resolved; EB—data analyses; revised the work for important intellectual content; final approval of the version to be published; agreement to be accountable for all aspects of the work in ensuring that questions related to the accuracy or integrity of any part of the work are appropriately investigated and resolved; PS—experimental design and organization; revised the work for important intellectual content; final approval of the version to be published; agreement to be accountable for all aspects of the work in ensuring that questions related to the accuracy or integrity of any part of the work are appropriately investigated and resolved; DX—experimental design and organization; revised the work for important intellectual content; final approval of the version to be published; agreement to be accountable for all aspects of the work in ensuring that questions related to the accuracy or integrity of any part of the work are appropriately investigated and resolved; PR—experimental design and organization, hypotheses, interpretation of data for the work; drafting the work, revised the work for important intellectual content; final approval of the version to be published, agreement to be accountable for all aspects of the work in ensuring that questions related to the accuracy or integrity of any part of the work are appropriately investigated and resolved.

## Funding

Brazilian Council for Scientific and Technological Development (CNPq), the Carlos Chagas Filho Rio de Janeiro State Research Foundation (FAPERJ), the National Institute of Science and Technology for Drugs and Medicines (INCT-INOFAR), the Department of Science and Technology (DECIT)/Ministry of Health, and the Coordination for the Improvement of Higher Level Personnel (CAPES). The funders had no role in study design, data collection, and analysis, decision to publish or preparation of the manuscript.

### Conflict of interest statement

The authors declare that the research was conducted in the absence of any commercial or financial relationships that could be construed as a potential conflict of interest.
